# Very low dislocation rate and good clinical outcome after Bereiter trochleoplasty and additional procedures following the Copenhagen patella–femoral instability algorithm: One‐ and two‐years outcomes from a consecutive cohort of 368 cases

**DOI:** 10.1002/ksa.12663

**Published:** 2025-03-25

**Authors:** Christian Dippmann, Volkert Siersma, Simone Rechter, Kristoffer W. Barfod, Peter Lavard

**Affiliations:** ^1^ Department of Orthopedic Surgery, Section for Sports Traumatology Copenhagen University Hospital Bispebjerg and Frederiksberg Copenhagen Denmark; ^2^ Department of Clinical Medicine University of Copenhagen Copenhagen Denmark; ^3^ Department of Public Health, The Research Unit for General Practice and Section of General Practice University of Copenhagen Denmark

**Keywords:** Bereiter trochleoplasty, patella dislocation, patello‐femoral instability, trochlear dysplasia

## Abstract

**Purpose:**

Patello–femoral instability (PFI) is often caused by predisposing factors, with trochlea dysplasia (TD) as the most prominent. Untreated patellar instability leads to impaired function and an increased risk of patellofemoral osteoarthritis. Since 2009 patients with PFI have been treated according to the Copenhagen PFI algorithm, where underlying osseous predisposing factors are addressed. The aim of this study was to report the two‐year outcome after Bereiter trochleoplasty (TP) for high grade TD in a cohort of 368 consecutive patients treated according to the Copenhagen PFI algorithm from 2011 to 2021.

**Methods:**

This was a register study evaluating a consecutive cohort of patients with high grade TD undergoing Bereiter trochleoplasty and additional procedures following the Copenhagen PFI algorithm. Outcomes were the Kujala score, the Knee Osteoarthritis Outcome Score (KOOS) and the Lysholm score collected preoperatively and after 1 and 2 years.

**Results:**

From January 2011 to September 2021, 368 Bereiter TPs were performed on 346 patients (99 males, 225 females and 44 bilateral surgeries). Four knees (1.1%) experienced a re‐dislocation. There were statistically significant (*p* < 0.0001) and clinically relevant improvements in all patients reported outcome measure (PROM)‐scores 1 and 2 years after surgery. Over 2 years Kujala score improved mean (95% confidence interval) 18.7 (16.5–20.9), KOOS QoL 31.0 (28.0–34.0) and Lysholm score 20.0 (17.1–22.9).

**Conclusions:**

Patients with high grade TD treated with Bereiter TP and additional procedures according to the Copenhagen PFI algorithm showed low re‐dislocation rate and statistically significant and clinically relevant improvement in patient reported outcome 1 and 2 years after treatment.

**Level of Evidence:**

Level II.

**Abbreviations**
95%CI: 95% confidence interval
CD index: Carton–Deschamps index
ET: Elmslie–Trillat
ICC: intra‐class correlation
IKDC: International Knee Documentation Committee
KOOS: Knee injury and Osteoarthritis Outcome Score
LoA: limits of agreement
LTI: lateral trochlea inclination
MCID: minimal clinical important difference
MDE: minimal detectable effect
MPFL‐R: medial patella‐femoral ligament reconstruction
PROM: patient reported outcome measure
PTI: patello–trochlea index
ROM: range of motion
SD: pre‐surgery standard deviation
TD: trochlear dysplasia
TP: trochleoplasty
TT: tibial tubercle transfer
TT‐TG: tibial tuberosity trochlea groove

## INTRODUCTION

Trochlea dysplasia (TD) is a major risk factor for recurrent patellar dislocation and patello–femoral instability (PFI) [6]. PFI leads to physical impairment [11] and increased risk of patello‐femoral cartilage lesions and osteoarthritis [29]. The risk of recurrent patellar dislocation increases in the presence of additional predisposing factors such as patella alta, lateralisation of the tibial tuberosity and/or valgus/torsional malalignment [1, 7, 8, 15, 21]. Due to its multifactorial nature, it is recommended that surgical treatment of TD is performed as a combined procedure, where relevant predisposing morphological factors are addressed. Trochleoplasty (TP) is the cornerstone in the treatment of high‐grade TD [14].

**Figure 1:**
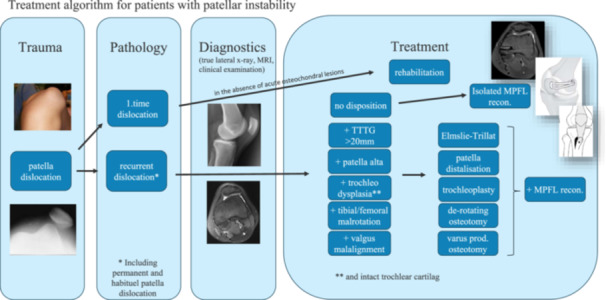
Treatment algorithm for patients with patella instability. Patients with recurrent dislocations, permanent or habitual patella dislocation will be examined clinically and by true lateral X‐ray, magnetic resonance imaging (MRI)+/‐computed tomography (CT) scan to identify all relevant predisposing factors. The following cut‐off values are used: TTTG > 20 mm on MRI scan for medialisation of the tibial tuberosity (Elmslie–Trillat), Carton–Deschamps (CD) index >1,2 or patella‐trochlea index (PTI) < 15% for distalisation of the tibial tuberosity, Dejour type B and D and lateral trochlea inclincation (LTI) < 11 degrees for trochleoplasty (TP), tibial torsion > 40° and/or femoral torsion > 35° for de‐rotating osteotomy and valgus angle > 10° for varus producing osteotomy.

In 2006, the Copenhagen PFI algorithm was developed as a treatment guide for decision‐making based on radiological and clinical criteria and since 2009 (Figure 1), all patients with recurrent PFI have been treated according to the algorithm in the eastern part of Denmark.

The aim of this study was to report the 2‐year outcome after Bereiter TP and additional procedures in patients with high grade TD according to the Copenhagen PFI algorithm from 2011 to 2021. We hypothesised that a low dislocation rate and good clinical outcome could be achieved using the extensive standardised treatment approach.

## MATERIALS AND METHODS

This was a register study evaluating a consecutive cohort of patients with high grade TD treated with TP. Since January 2011 patients have been prospectively followed with registration of preoperative, perioperative and postoperative data 1, 2, 5 and 10 years after TP. This is a presentation of the two‐year data. At initiation of the register no IRB approval was needed according to Danish legislation. Institutional Review Board approval for analysis and publication of data was achieved in 2020 from the data registration committee (P‐2020‐573) and the ethical committee (Journal‐no: H‐20031969) of the Capital Region of Denmark.

### Patients

**Figure 2:**
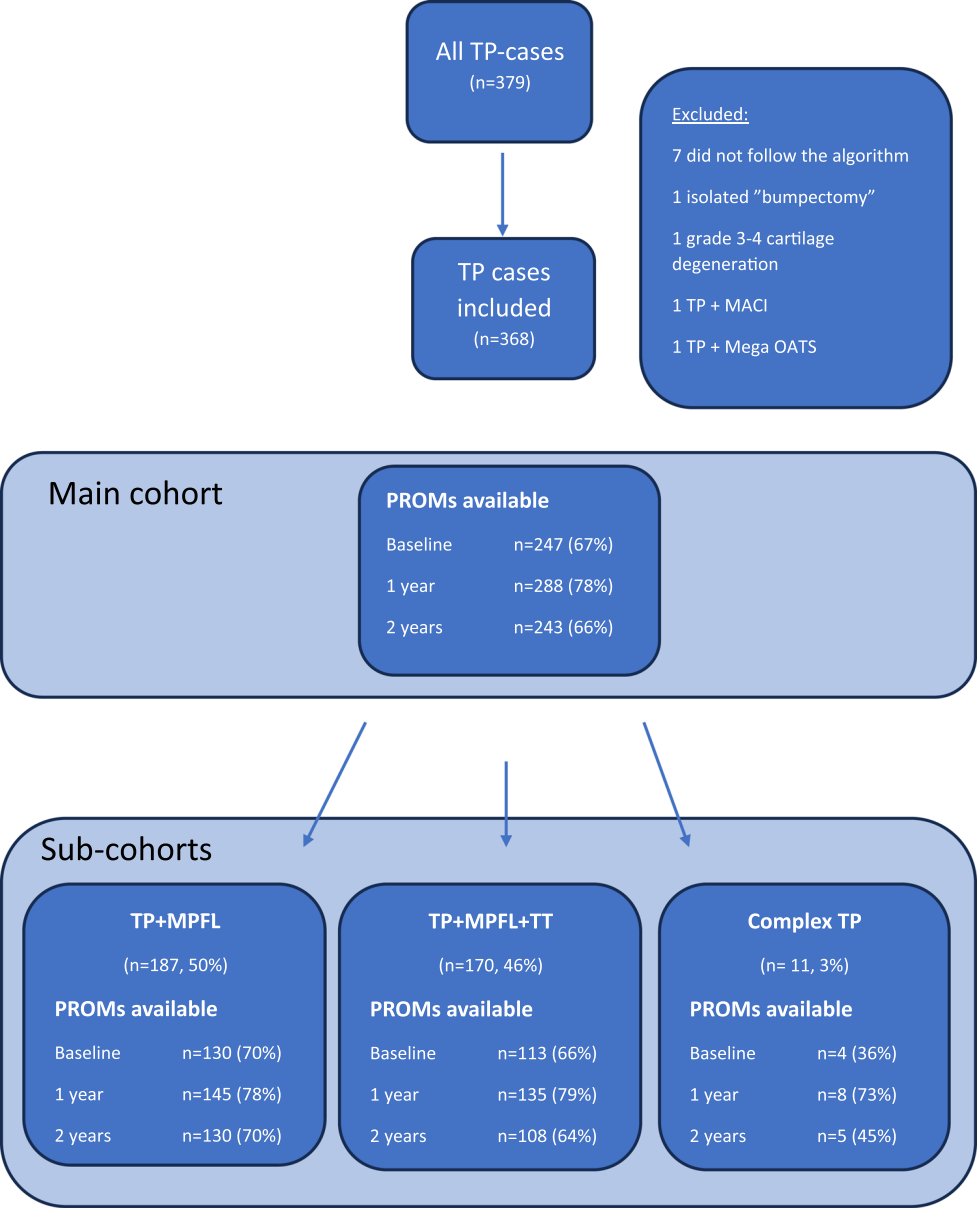
Patient flowchart describing the patients included in the cohort, procedures performed and availability of PROM at baseline, 1 and 2 years follow up. MACI, matrix‐induced chondrocyte implantation; MPFL, medial patello–femoral ligament; OATS, osteochondral autograft transplantation; PROMs, patients reported outcome measures; TP, trochleoplasty; TT, tibial tubercle transfer.

A consecutive cohort of patients treated with TP from 2011 to 2021, covering 78% of all TPs in Denmark, were included. Patients were selected for TP according to the Copenhagen PFI algorithm patients not treated according to the algorithm were excluded as were patients with cartilage degeneration ICRS Grade 3–4, and patients receiving advanced cartilage procedures (Figure 2).

#### Indication for TP according to the Copenhagen PFI algorithm

According to the Copenhagen PFI treatment algorithm, patients with recurrent patellar instability were examined with true lateral X‐ray and an MRI of the affected knee. In case of clinical suspicion of coronal plane or valgus malalignment long leg standing X‐rays and/or rotational CT‐scan of the hip, knee and ankles was performed.

TP was performed and patients included in the cohort with (1) Dejour v.2 [32] Class B or D, in combination with a lateral trochlea inclination (LTI) [6] measured with a modified two‐image technique below 11° [6, 9]. (2) Skeletal maturity with closed epiphysis in the femur and tibia evaluated using an AP and lateral radiograph of the knee. There was no upper age limit for TP. (3) Patients with osteoarthritic changes in the femoral cartilage Kellgren Lawrence Grade 4 were not considered candidates for TP and as such excluded from entering the cohort. TP was always combined with a reconstruction of the medial patello‐femoral ligament (MPFL‐R) using a gracilis tendon autograft in primary and quadriceps tendon autograft in secondary MPFL‐R [31].

Indication for additional procedures following the Copenhagen PFI algorithm were as followed: Medialisation of the tibial tuberosity was performed in patients with tibial tuberosity trochlear groove (TT‐TG) distance [31] exceeding 20 mm on an axial MRI scan. Distalisation of the tibial tuberosity was performed in patients with patella alta, defined by Caton–Deschamps index > 1.2 [4]. Since 2020 measurement of the patella–trochlea index (PTI) was added and values < 15% were considered pathological [2]. Since 2016 the Copehagen PFI algorithm was extended including axial and frontal malalignment. De‐rotational osteotomy was performed in patients with increased femoral anteversion exceeded 35° and/or increased external rotation of the tibia, exceeded 40°. Rotational malalignment was measured using whole leg CT scans, according to Waidelich et al. [34]. Varus producing femoral osteotomy was considered if knee valgus exceeds 5° on a full length, standing radiograph [5].

### Surgical technique

**Figure 3:**
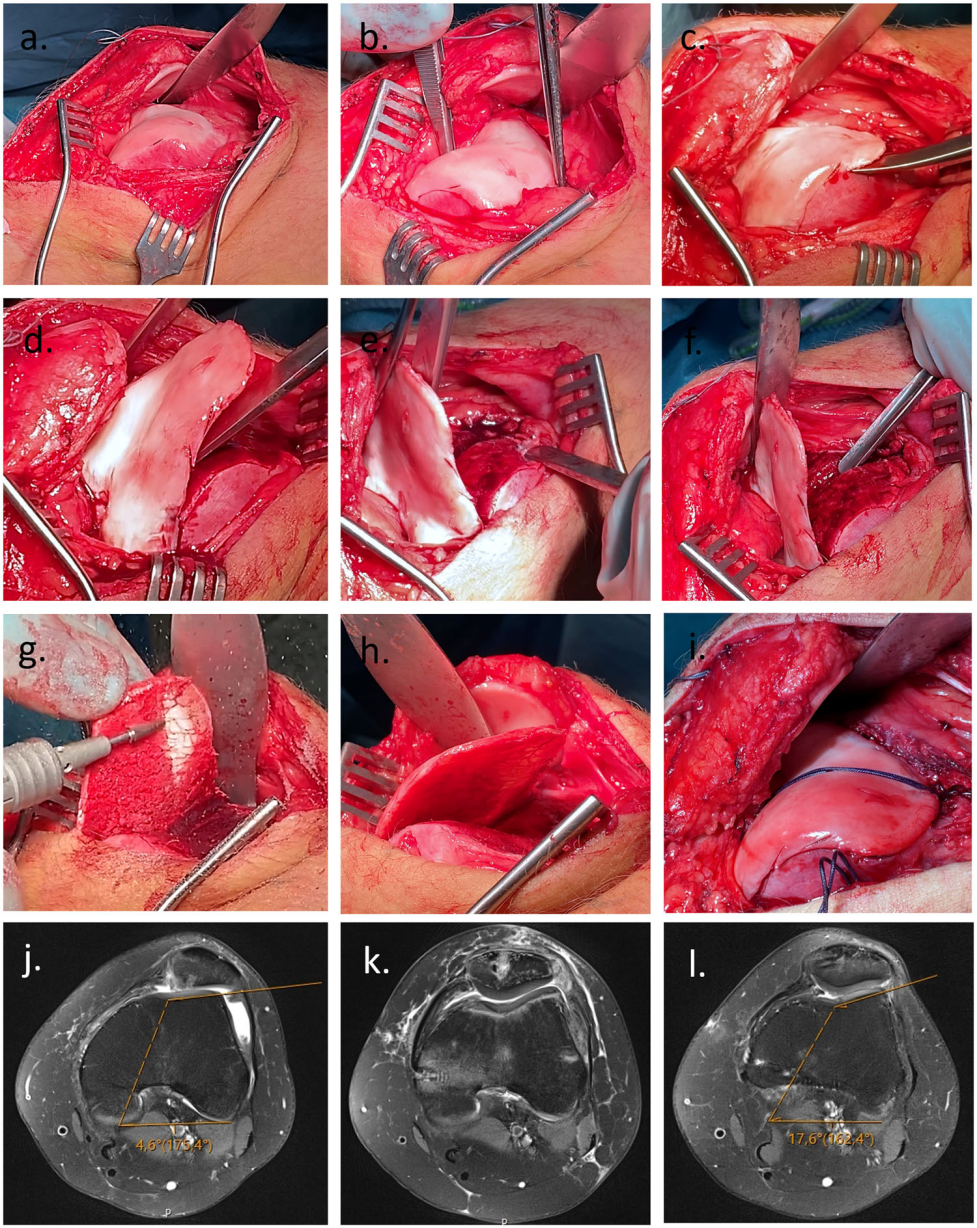
Surgical pictures of Bereiter trochleoplasty (TP). First the trochlea is exposed through a lateral approach (a) and the direction of the new groove evaluated (b). A thin cartilage flap is release from the femoral trochlea (c, d) and the new trochlea groove is created using straight and curved chisels (e, f) and a highspeed burr (g) keeping as much height as possible on the lateral facet of the trochlea to normalise the inclination. The shape of the patellar joint surface is also considered when the trochlear groove is created to secure patello‐femoral congruency. The cartilage flap is modelled into the groove (e) and fixed centrally with a resorbable suture size 6 through two drillholes to the lateral side of the femur and tied (f). The increase of the lateral trochlea inclination (LTI) from pre‐ (g) to 2 weeks and 52 weeks postoperatively (h, i) can be seen on an axial magnetic resonance imaging (MRI) scan.

The knee was approached through a midline skin incision (Figure 3). The joint was opened through a lateral arthrotomy. If a distalisation of the tibial tuberosity was planned, the osteotomy was performed prior to the TP. The osteochondral trochlea flap was raised using straight and curved osteotomes and was thinned, leaving a 1–2 mm bone layer. The focus in performing a TP was achieve patella–femoral alignment and to ensure as much height of the lateral facet as possible. The cartilage flap was modelled into the groove and fixed centrally with a reabsorbable 10 mm Vicryl tape (Ethicon, Johnson & Johnson), which was tied at the lateral femur after being passed through two bone tunnels. The vastus lateralis tendon was reattached, and if a tibial tuberosity transfer (medialisation and/or distalisation) was planned, this was carried out before performing the medial patello‐femoral ligament reconstruction (MPFL‐R). The tibial tuberosity transfer was secured by two 4.5 mm cannulated screws. Finally, the MPFL‐R was performed, using a gracilis autograft which was looped inside the proximal half of the patella through converging drillholes and fixed with a bio‐interference screw at the Schoettle point, identified by fluoroscopy [31]. Passive ROM was tested to ensure proper tension and isometry of the MPFL‐R, and the graft was fixed with an interference screw. The lateral capsulotomy was left open while subcutis and the skin was closed separately. In cases of valgus malalignment a lateral open wedge distal femoral osteotomy or a medial closed wedge high tibial osteotomy was performed, depending on the anatomical axis. In cases with rotational malalignment a distal femoral de‐rotating osteotomy and/or a proximal high tibial osteotomy was performed [5].

#### Post‐operative regime

Post‐operatively, the knee was immobilised in a hinged brace at 30° flexion for 2 weeks, allowing 50% weight bearing. Weeks 3–6 range of motion (ROM) 0–90 and weight bearing as tolerated was allowed, unless an additional frontal or axial osteotomy was added, in which case weightbearing was reduced to 50% for 6 weeks. Patients were discharged the day after surgery. Pain was managed with single‐shot femoral nerve block and oral pain killers. Thrombosis prophylaxis (Tinzaparin 3500IE) was given for 2 weeks. Patients were referred to a 12‐week supervised rehabilitation course free of charge. If ROM 0°–120° was not achieved within the first 3 months, arthroscopically assisted mobilisation (AAM) with removal of adhesions and passive mobilisation was performed.

#### Subdivision of the cohort

The main cohort including all patients treated with TP and MPFL‐R was subdivided into the following groups: (1) Patients treated with TP and MPFLR without additional procedures, (2) patients with TP and MPFL‐R with additional tibial tubercle transfer (TT) and (3) complex TP‐cases, who underwent TP, MPFL‐R and TT with additional frontal and/or de‐rotational aligning surgery.

### Data collection

Data were collected preoperatively, perioperatively and 1 and 2 years after TP. Sex, age, clinical, radiological and patient‐reported data was collected from the database. Information regarding complications and additional surgical procedure prior to TP and in the 2 years follow‐up period after TP was collected from the electronic patient journal (Epic®). Additionally all patients were invited to a 2 years follow‐up consultation to ensure completeness of all relevant clinical data.

### Outcomes

Treatment effect was evaluated investigating re‐dislocation rate, PROM and complications. Patellar re‐dislocation was defined as complete dislocation of the patella and was registered throughout the hole study period.

Patient reported outcome was reported using the Kujala score [17], the Knee injury and Osteoarthritis Outcome Score (KOOS) [27] and the Lysholm score [20].

Complications were defined as adverse events such as infection, deep vein thrombosis, development of a post‐operative stiff knee, subsequent surgery of the index knee, including arthroscopically assisted manipulation in general anaesthesia due to a stiff knee and revision surgery due to infection or persisting patella‐femoral instability.

### Statistical analysis

Descriptive statistics of the main cohort and the subgroups was performed according to the nature of data. Mean difference in PROM from baseline to follow up were estimated using mixed models featuring a patient random effect to adjust the variance for repeated observations on the same person. These estimates were adjusted for patient age, whether there was previous surgery, and whether there was a subsequent surgery after TP. Significance level was set at 0.05 and 95% confidence intervals (95%CIs) were reported. No minimal clinically important difference (MCID) has been established for the PROM scores for patients with PFI, why a change of 50% of the preoperative standard deviation (SD) was recognised as a useful threshold for patient‐perceived change between preoperative and postoperative scores [19].

## RESULTS

**Figure 4:**
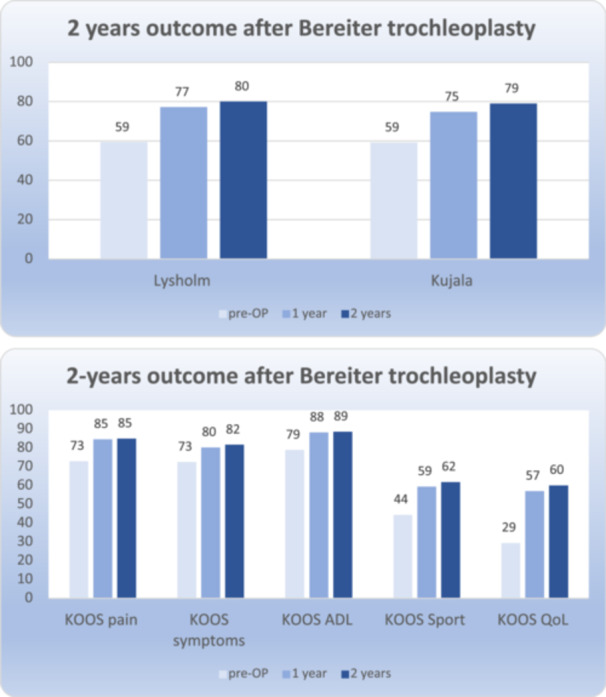
Significant improvement in all PROM‐scores can be seen 1, 2 and 5 years after patella stabilising surgery with Bereiter trochleoplasty. ADL, activities of daily living; KOOS, Knee Osteoarthritis Outcome Score; PROM, patients reported outcome measure; QoL, quality of life.

During the study period (2011–2021) 368 Bereiter TPs were performed on 346 patients (99 males, 225 females and 44 bilateral surgeries (Table 1, Figure 4). PROM data completeness for the main cohort was 67% for pre‐operative, 78% for 1‐year and 66% for 2‐year PROMS (Figure 2). A total of 83% of all patients attended their 2‐years follow‐up consultation to ensure completeness of for patella dislocation and/or subsequent surgery performed at other institutions.

**Table 1:** Patient characteristics, procedures performed, subsequent surgery, complications and data completeness.

Patient characteristics	
Cases	368 Knees (346 patients)
Side	
Left	174
Right	150
Bilateral	44
Age	22 years (12–47years)
Sex	
Female	225
Male	99
Procedures performed	
Trochleoplasty with MPFL‐R	368
Elmslie–Trillat	90 (24%)
ET + Tibial tuberosity distalisation	55 (15%)
Combined procedures (TP+MPFL‐R) +	11 (3%)
Vastus lateralis plasty	5
De‐rotating DFO	3
De‐rotating DFO	1
De‐rotating DFO	1
Varus producing DFO + calcaneus osteotomy	1
Subsequent surgery (in total)	101 (27%)
Arthroscopically assisted mobilisation	49 (13%)
Hardware removal	16
Hardware removal	13
Knee arthroscopy	6
Revision MPFL‐R	6
Revision MPFL+ET	2
Wound revision	2
Cosmetic scar revision	2
Prophylactic fasciotomy	1
Vastus lateralis reinsertion	1
Complication	
Re‐dislocation	4 (1.1%)
Major complications *(deep vein thrombosis, infection, revision TP, etc.)*	0

Abbreviations: DFO, distal femur osteotomy; ET, Elmslie–Trillat; MPFL‐R, medial patellofemoral ligament reconstruction; OATS, osteochondral autologous transplantation; TP, trochleoplasty; TT, tibial tuberosity.

### Re‐dislocation

Four knees (1.1%) experienced a re‐dislocation within 2 years.

### Patient reported outcomes

There were considerable improvements in all PROM scores from baseline to 1 and 2 years (*p* < 0.0001) for the main (Table 2) and the sub‐cohorts (Table 3). The Kujala score improved mean (95%CI) 15.2 points (13.3–17.4) after 1 year and 18.7 points (16.5–20.9) after 2 years. Lysholm improved 17.6 points (14.8–20.5) after 1 year and 20.0 points (17.1–22.9) after 2 years. KOOS subscales improved between 9.1 and 31.0 points with the most predominant improvement in KOOS QoL with 27.7 points (24.8–30.6) after 1 year and 31.0 points (28.0–34.0) after 2 years. When subdividing the main cohort in the three sub‐cohorts: TP + MPFL‐R, TP + MPFL‐R + TT and TP + MPFL‐R + TT with additional procedure (complex), no difference in mean improvement between TP + MPFL‐R and TP + MPFL‐R + TT can be seen. The complex TP cases, where frontal and/or axial alignment surgery was added, reported a high mean improvement in KOOS QoL.

**Table 2:** Patient reported outcome measures for all patients undergoing TP at baseline and 1 and 2 years after surgery.

	Baseline			1 year				2 years			
	0.5 SD	Mean	*n*	Mean	*n*	Mean diff (95%CI)	Sign	Mean	*n*	Mean diff (95%CI)	Sign
Kujala	8.7	5.2	229	74.9	265	15.4 (13.2–17.5)	<0.0001	78.7	232	18.9 (16.6–21.1)	<0.0001
KOOS pain	9.5	72.9	226	84.7	268	11.7 (9.5–13.9)	<0.0001	85.1	230	12.0 (9.7–14.3)	<0.0001
KOOS symp	9.3	72.5	227	80.4	268	7.8 (5.6–10.0)	<0.0001	81.9	228	9.3 (7.0–11.6)	<0.0001
KOOS ADL	9.9	78.8	227	88.2	268	9.6 (7.1–12.0)	<0.0001	88.6	229	9.8 (7.2–12.3)	<0.0001
KOOS sport/recreation	14.3	44.3	221	59.6	266	15.7 (12.1–19.3)	<0.0001	62.4	229	18.7 (15.0–22.5)	<0.0001
KOOS QoL	8.5	29.4	226	57.3	267	27.9 (25.0–30.8)	<0.0001	60.3	228	31.1 (28.1–34.1)	<0.0001
Lysholm	9.6	59.4	132	77.5	190	17.9 (15.1–20.8)	<0.0001	80.3	186	20.1 (17.2–23.1)	<0.0001

Abbreviations: ADL, activities of daily living; CI, confidence interval; KOOS, Knee Osteoarthritis Outcome Score; QoL, quality of life; SD, standard deviation.

**Table 3:** Patient reported outcome measures (PROMs) at baseline, and 1 and 2 years after surgery, stratified for TP+MPFL‐R, TP+MPFL‐R+TT and “complex” cases (TP+MPFL‐R+TT+additional procedures).

	Baseline		1 year				2 years				
	Mean	*n*	Mean	*n*	Mean diff (95%CI)	Sign	Mean	*n*	Mean diff (95%CI)	Sign	Int
TP+MPFL											
Kujala	58.4	122	73.7	132	14.8 (11.8–17.7)	<0.0001	77.9	121	19.0 (16.0–22.1)	<0.0001	0.9688
KOOS pain	70.9	120	83.2	131	12.0 (8.9–15.1)	<0.0001	82.9	121	11.9 (8.7–15.1)	<0.0001	0.9518
KOOS symp	70.1	120	79.4	131	8.6 (5.5–11.7)	<0.0001	80.8	119	10.2 (7.0–13.4)	<0.0001	0.8434
KOOS ADL	77.9	120	87.8	131	9.7 (6.3–13.2)	<0.0001	86.6	120	8.9 (5.4–12.4)	<0.0001	0.8908
KOOS sport/recreation	42.9	116	59.2	129	15.2 (10.1–20.3)	<0.0001	63.0	120	20.1 (14.9–25.3)	<0.0001	0.8174
KOOS QoL	30.0	119	56.7	130	26.7 (22.6–30.7)	<0.0001	58.8	120	28.3 (24.2–32.4)	<0.0001	0.2081
Lysholm	60.0	71	75.5	93	15.6 (11.6–19.5)	<0.0001	79.2	97	18.6 (14.6–22.5)	<0.0001	0.4297
TP+MPFL+TT											
Kujala	60.2	101	75.9	128	15.9 (12.7–19.0)	<0.0001	79.4	105	18.7 (15.3–22.0)	<0.0001	
KOOS pain	74.8	101	85.9	131	11.5 (8.2–14.7)	<0.0001	87.1	103	12.3 (8.9–15.8)	<0.0001	
KOOS symp	75.2	102	81.4	131	6.9 (3.7–10.1)	<0.0001	83.4	103	8.5 (5.0–11.9)	<0.0001	
KOOS ADL	79.7	102	88.4	131	9.1 (5.5–12.7)	<0.0001	90.8	103	10.7 (6.8–14.5)	<0.0001	
KOOS sport/recreation	45.1	101	59.4	131	16.3 (11.1–21.6)	<0.0001	61.4	103	17.4 (11.8–23.1)	<0.0001	
KOOS QoL	28.7	102	57.4	131	28.6 (24.5–32.8)	<0.0001	61.4	102	33.7 (29.2–38.2)	<0.0001	
Lysholm	57.7	57	79.1	93	21.1 (16.8–25.4)	<0.0001	81.0	84	22.5 (18.0–26.9)	<0.0001	
Complex TP											
Kujala	60.0	6	81.4	5	16.5 (2.3–30.7)	0.0225	82.7	6	18.5 (4.7–32.4)	0.0089	
KOOS pain	82.4	5	91.3	6	6.3 (−8.5 to 21.1)	0.4029	95.7	6	8.4 (−7.0 to 23.8)	0.2840	
KOOS symp	76.0	5	79.7	6	3.9 (−10.9 to 18.7)	0.6053	77.8	6	2.9 (−12.5 to 18.3)	0.7130	
KOOS ADL	80.2	5	93.7	6	13.3 (−3.2 to 29.7)	0.1142	92.7	6	12.0 (−5.1 to 29.0)	0.1678	
KOOS sport/recreation	61.3	4	71.3	6	7.4 (−19.2 to 34.0)	0.5856	67.5	6	10.5 (−16.1 to 37.1)	0.4368	
KOOS QoL	28.6	5	67.7	6	42.1 (22.9 to 61.3)	<0.0001	72.0	6	46.1 (26.1–66.1)	<.0001	
Lysholm	72.5	4	89.5	4	13.9 (−3.3 to 31.1)	0.1136	90.0	5	15.2 (−2.9 to 33.2)	0.0987	

Abbreviations: ADL, activities of daily living; CI, confidence interval; KOOS, Knee Osteoarthritis Outcome Score; QoL, quality of life; MPFL‐R, medial patellofemoral ligament reconstruction; TP, trochleoplasty; TT, tibial tuberosity.

### Complications

There were no major complications within the first 2 years after surgery. Ten minor complications (seven surgical and three non‐surgical) were registered.

### Surgical complications

One patient suffered from vastus lateralis rupture under postoperative mobilisation and underwent secondary reinsertion. One patient underwent a postoperative fasciotomy due to clinical suspicion of an acute anterior compartment syndrome, which could not be confirmed intraoperatively. Two patients underwent extra‐articular surgical revision, one had a subcutaneous haematoma due to an undiagnosed haemophilia, while one patient underwent scar revision due to a reaction towards the suture material. Furthermore, two patients had surgical revision due to cosmetic reasons, and one patient suffered from a post‐operative MPFL‐pull out at the day of surgery and underwent subsequent re‐fixation. *Non‐surgical complication*: Three cases of superficial infection, which were treated with oral antibiotics.

Subsequent surgery was performed in 27% (102 knees), involving arthroscopic release and brisement force for limited ROM in 13% (48 cases) as the largest subgroup (Table 1). In 75% (27 cases) a ROM normalisation was achieved. In total eight patients had to undergo revision MPFL reconstruction. The indication for revision MPFL was ongoing pain (3), instability without patellar dislocation (3) and patellar dislocation (2). Six cases had isolated MPFL‐R, while a combined MPFL‐R with tibial tuberosity medialisation was performed in two cases. Hence, five patients (1.4%) underwent revision due to PFI.

## DISCUSSION

The most important finding of the present study was, that patients with high grade TD and recurrent patella instability had a very low re‐dislocation rate and statistically significant improvement in patient reported outcome 1 and 2 years after Bereiter TP. Following the Copenhagen PFI algorithm, where relevant predisposing factors are addressed in a single procedure a low complication‐ and re‐operation rate due to PFI could be seen.

### Dislocation

As recurrent patellar dislocation is the main indication for patella stabilising surgery, the re‐dislocation rate can be considered the most important singular outcome factor. In this consecutive cohort of 368 cases only four patients (1.1%) experienced patella re‐dislocation. In a meta‐analysis reporting the re‐dislocation rate after Bereiter TP [28], the weighted rate was 4%, ranging from 0% to 21%. Reporting the short‐term outcome, one could argue, that the low re‐dislocation rate in the present cohort may be biased as patients not yet have regained full pre‐surgical activity levels. However, two years after surgery full return to activity is expected and significant improvement is seen in all PROMS with a pronounced improvement in the KOOS domains “KOOS sport” and “KOOS QoL”, which we believe is indicative that patients have regained or even increased their activity level compared to baseline.

### Outcome

Generally, Bereiter TP yields good clinical results [22, 24, 25, 26, 33]. However, most studies report short‐term outcome (<3 years) [9, 12, 23, 35] or mean mid‐term outcomes between 2 and 5 years [9, 23, 26, 33]. In this study significant improvement can be seen in all PROMS (Kujala, Lysholm and KOOS) 1 and 2 years after surgery. Additionally, the main cohort has been subdivided to analyse whether the complexity of the surgery negatively influences the outcome. When comparing mean improvement for patients undergoing ‘simple’ TP and MPFL‐R with patients undergoing TP with MPFL‐R and TT similar improvements were observed at 1 and 2 years post‐operatively. The third subgroup of the most complex cases, which required frontal or/and axial alignment surgery reported a more pronounced mean improvement in KOOS QoL than the other two sub‐groups. However, as the PROM data of this subgroup is rather small (*n* = 6), these results must be interpreted with care. Although statistically significant improvements can be seen in all scores, it is unclear if these improvements represent clinical improvements too. In the meta‐analysis by Zaffagnini et al. [36] a mean difference of 28.1 points in the Kujala score is suggested as a clinically relevant improvement after TP with MPFL reconstruction which is almost the double of the improvements seen in this study, being 15.2 after 1 year and 18.7 after 2 years. In other studies investigating effect of TP the post‐operative improvement in Kujala score varies between 17 [24] and 22 points [12]. However, as stated by Leclerc et al. [18], the heterogeneity of the current literature and the variation of the outcome measures, makes direct comparison difficult. The prerequisite for establishing a MCID is that the PROM is reasonable valid. Kujala, KOOS and Lysholm score had not been developed specifically for patients with patellofemoral instability, and patients had not been involved in the wording and selection of items [13]. In addition, MCID must be calculated for the specific follow‐up period, as it is not necessarily the same at different time points. Therefore, it is difficult to compare the improvements that are described after surgery in the different series. MCID calculated by a distribution‐based method is defined as a change of more than 0.5 SD. For the current cohort MCID would therefore be between 7.5 and 10 points—and the improvements that were observed are well beyond that.

### Complications

In this study none of the patients suffered from major complications such as deep vein thrombosis, intraarticular infection, or revision TP. Minor complications such as soft‐tissue infections, post‐operative joint stiffness, and subsequent surgery due to pain and subjective instability are reported.

Historically, a residual or even increased post‐TP pain [16] has been described [10]. Recent literature does not seem to support that fact as several studies have shown decreased post‐operative pain levels measured by Kujala and KOOS pain after successful Bereiter TP [9, 23, 26, 33]. In line with the results presented in this study (Table 2), pain levels can in general be expected to decrease [23, 37].

Overall, 109 patients (27%) underwent subsequent surgery, mainly due to the surgical treatment of a post‐operative stiff knee by arthroscopically assisted manipulation (AAM) (13%).

In their meta‐analysis on complications following TP, Leclerc et al. [18] reported an overall rate for post‐operative joint stiffness of 3%. In the current study, an initially increased rate of subsequent surgery due to post‐operative joint stiffness of 13% can be seen, which is reduced to 5.6% after AAM treatment. If this can be explained by a more aggressive approach with early AMM within 12 weeks or an increased soft tissue trauma due to the high number of combined procedures (51%), remains unclear.

According to Leclerc et al. [18] residual PFI is another common complication in Bereiter TP (6%), leading to rate of subsequent surgery of 11%. In this study, subsequent surgery due to residual PFI was performed in five cases (1,4%).

Subsequent surgery rates vary among different trochlea re‐modelling techniques, ranging from 11.1% for Bereiter TP to 57.8% for wedge osteoplasty [18]. As it has been shown that the amount of subsequent surgery increases in studies with longer follow‐up [18], the rate of subsequent surgery must be put in relation to the study period. In their studies analysing the long‐time (>53years) outcome after Bereiter TP, Metcalf et al. [23] and Wind et al. [35] report rates of subsequent surgery of 27% and 14%, while Bering et al. and Camathias et al. [3] report a rate of 5% and 10% respectively in a medium‐term outcome.

A 27% rate of subsequent surgery is high but considering the extend of the surgery it probably expectable when a thorough review of operation registers and patient journals is performed. A study investigating the risk of reoperation after primary repair of the anterior cruciate ligament found up to 24% reoperation rate within the first year when performing thorough review of operation registers and patient journals [30]. While treatment of residual instability, treatment of a stiff knee or soft tissue revision can be considered a complication, metal removal, knee arthroscopy due to cartilage degeneration or PF stabilising surgery due to a new trauma might not.

### Strengths and limitations

The strength of the study is the prospectively collected data and the almost complete information on patient history, re‐operation and re‐dislocation. The Copenhagen PFI algorithm has been used for over 10 years with only a small alteration related to the diagnosis of rotational alignment.

While the completeness of PROM data in the literature is varying between 42% and 77% [22, 25, 33], completeness for PROM data in the present study is 67% (*n* = 242) for pre‐operative PROMS, 78% (*n* = 285) for 1‐year PROMS and 66% (*n* = 232) for 2‐year PROMS. 83% (*n* = 311) of all patients attended their 2‐year follow‐up, while electronic patient journals of all patients were reviewed by the time of data analysis.

The study is limited by the lack of a control group making it impossible to conclude if the seen improvements are due to the surgical treatment an unrecognised confounding or merely regression towards the mean. Also, the lack of a valid PROM for PFI patients is a concern [13], but the improvements reflected in the non‐specific PROM‐scores indicates an actual improvement of the subjective condition as the non‐specific scores are less likely to show a difference compared to specific PROM scores [13]. Finally, the results are not necessarily generalisable to other TP treatment regimens.

## CONCLUSION

Patients with high grade TD treated with Bereiter TP according to the Copenhagen PFI algorithm showed low re‐dislocation rate and statistically significant improvement in patient reported outcome one and two years post‐operatively.

## AUTHOR CONTRIBUTIONS

Christian Dippmann and Peter Lavard conceptualised and designed the study. Simone Rechter, Peter Levard and Christian Dippmann performed the data acquisition. Volkert Siersma performed the statistical analysis and provided statistical consultation. Volkert Siersma, Kristoffer Barfod and Christian Dippmann wrote and revised the manuscript. All authors reviewed the manuscript.

## CONFLICT OF INTEREST STATEMENT

The authors declare no conflicts of interest.

## ETHICS STATEMENT

Ethical approval was not needed for the present study. Formal consent was given by all participating patients.

## Data Availability

All data generated or analyse during this study are included in this published article.
